# Diagnostic accuracy of an integrated respiratory guideline in identifying patients with respiratory symptoms requiring screening for pulmonary tuberculosis: a cross-sectional study

**DOI:** 10.1186/1471-2466-6-22

**Published:** 2006-08-25

**Authors:** René G English, Max O Bachmann, Eric D Bateman, Merrick F Zwarenstein, Lara R Fairall, Angeni Bheekie, Bosielo P Majara, Carl Lombard, Robert Scherpbier, Salah Eddine Ottomani

**Affiliations:** 1 Knowledge Translation Unit, University of Cape Town Lung Institute, George Street, Mowbray, 7700, Cape Town, South Africa; 2 Department of Health Policy and Practice, School of Medicine, University of East Anglia, Norwich, NR47TJ, UK; 3 Knowledge Translation Programme and Department of Health Policy, Management and Evaluation, Faculty of Medicine, University of Toronto, Ontaria, M56IV7, Canada; 4 School of Pharmacy, University of the Western Cape, Bellville, 7535, Cape Town, South Africa; 5 Department of Community Health, University of the Free State, Bloemfontein, South Africa; 6 Biostatistics Unit, Medical Research Council, Tygerberg, Cape Town, South Africa; 7 Stop TB, World Health Organization, Geneva, Switzerland

## Abstract

**Background:**

To evaluate the diagnostic accuracy of the integrated Practical Approach to Lung Health in South Africa (PALSA) guideline in identifying patients requiring bacteriological screening for tuberculosis (TB), and to determine which clinical features best predict suspected and bacteriologically-confirmed tuberculosis among patients with respiratory symptoms.

**Methods:**

A prospective, cross-sectional study in which 1392 adult patients with cough and/or difficult breathing, attending a primary care facility in Cape Town, South Africa, were evaluated by a nurse using the guideline. The accuracy of a nurse using the guideline to identify TB suspects was compared to that of primary care physicians' diagnoses of (1) suspected TB, and (2) proven TB supported by clinical information and chest radiographs.

**Results:**

The nurse using the guideline identified 516 patients as TB suspects compared with 365 by the primary care physicians, representing a sensitivity of 76% (95% confidence interval (CI) 71%–79%), specificity of 77% (95% CI 74%–79%), positive predictive value of 53% (95% CI 49%–58%), negative predictive value of 90% (95% CI 88%–92%), and area under the receiver operating characteristic curve (ARUC) of 0.76 (95% CI 0.74–0.79). Sputum results were obtained in 320 of the 365 primary care physicians TB suspects (88%); 40 (13%) of these were positive for TB. Only 4 cases were not identified by the nurse using the guideline. The primary care physicians diagnostic accuracy in diagnosing bacteriologically-confirmed TB (n = 320) was as follows: sensitivity 90% (95% CI 76%–97%), specificity 65% (95% CI 63%–68%), negative predictive value 7% (95% CI 5%–10%), positive predictive value 99.5% (95% CI 98.8%–99.8%), and ARUC 0.78 (95% CI 0.73–0.82). Weight loss, pleuritic pain, and night sweats were independently associated with the diagnosis of bacteriologically-confirmed tuberculosis (positive likelihood ratio if all three present = 16.7, 95% CI 5.9–29.4).

**Conclusion:**

The PALSA guideline is an effective screening tool for identifying patients requiring bacteriological screening for pulmonary tuberculosis in this primary care setting. This supports the randomized trial finding that use of the guideline increased TB case detection.

## Background

Tuberculosis (TB) has been declared a global emergency,[1] with the incidence rising by one percent each year [2]. In 2003 the global TB case-detection rate was only 45%, falling short of the global target of 75% [3]. One reason cited for the under-detection of TB in high-prevalence areas has been the failure of healthcare workers to consider TB in the differential diagnosis of patients with respiratory symptoms, resulting in diagnostic delays [4-6].

The Practical Approach to Lung Health in South Africa (PALSA) initiative is the South African adaptation of the World Health Organisation's (WHO) Stop-TB Practical Approach to Lung Health (PAL) strategy [7]. It was developed to improve the detection and management of common respiratory diseases, including TB, through the integration of vertical respiratory programmes in resource-poor settings. In South Africa nurses provide most primary care and often have limited physician support [8]. Many are inadequately skilled and equipped for accurate diagnosis [9], which includes distinguishing between several respiratory diseases with similar clinical presentations. PALSA therefore focuses on enabling primary nurse care practitioners to function as frontline clinicians through the provision of an evidence-based guideline that integrates TB with other common respiratory diseases such as asthma, COPD, and respiratory tract infections. The guideline identifies syndromes using symptoms and signs that best predict each disease (see Additional file 1).

Guidelines have been shown to be effective if tailored to local circumstances and practices [10]. The PALSA guideline therefore considers conditions in local primary healthcare facilities and is consistent with national drug and health department policies. Additionally, it was developed in consultation with various stakeholders and key roleplayers to encourage local ownership. This is the first documented attempt to integrate respiratory diseases into a single syndromic guideline in South Africa.

The effectiveness of a clinical practice guideline designed to change professional practice is best evaluated by means of a pragmatic randomised controlled trial, as we have done before [11]. In this study, we aimed to evaluate how accurately the integrated algorithmic PALSA guideline detected respiratory diseases when applied to patients with cough and/or difficult breathing. We therefore determined the diagnostic accuracy of the guideline, as used by a nurse, to identify patients requiring bacteriological screening for TB. The two reference standards were (1) suspicion of pulmonary TB by a primary care physican using clinical judgment about symptoms, signs and radiology results (without the results of sputum tests), and (2) diagnosis of pulmonary TB by a primary care physician using clinical judgment about symptoms, signs and radiology results, with confirmation by bacteriological testing of TB suspects. A second objective was to identify the clinical variables that best predicted patients requiring screening for TB, and patients with bacteriologically-confirmed TB, in this setting.

## Methods

### The guideline

The PALSA guideline [see Additional file 1] was developed over a period of one year by a working group comprising respiratory specialists, primary care and public health practitioners, a pharmacist, and health systems researchers. Focus group sessions with nurses, primary care doctors, health department managers and local experts elicited their practices, needs, and views on the content and practicality of the guideline. National policies and programmes such as the South African National Tuberculosis Control [12] and Essential Drugs List [13] programmes were adhered to. Literature was reviewed to determine the individual and combination of symptoms and signs that best predicted each respiratory condition or syndrome.

The criteria for entry into the study were cough and/or difficult breathing in patients aged 15 years and older. In the algorithm, the presence of these symptoms for 2 weeks or longer distinguished chronic from acute diseases. Features of a chronic presentation that prompted the need for screening for TB included cough and/or difficult breathing for more than 2 weeks with or without fever, pain on breathing or coughing, sputum production, unexplained weight loss, loss of appetite, night sweats, fever, blood-stained sputum or frank haemoptysis, and/or known positive human immunodeficiency virus (HIV) test or clinical acquired-immunodeficiency syndrome (AIDS). Features of an acute presentation considered to require screening for TB were cough and/difficult breathing for less than 2 weeks in patients with fever, pain on breathing or coughing, and/or sputum production. To encourage screening for TB, the algorithm reminds users to consider tuberculosis at several decision points. Instructions in the algorithm advise on the selection and timing of investigations, referral and follow up. When TB is suspected, the instruction is to send sputum for bacteriological testing. In the current validation study, however, the primary care physicians rather than the nurse using the guideline requested sputum tests on patients they considered to require bacteriological screening for TB.

### Patient population

The study was conducted at a large primary care clinic in Cape Town, South Africa, from November 2002 to December 2003. This centre is attended by approximately 9000 patients each month, and provides a 24-hour accident and an emergency service, and an outpatient clinic that operates on weekdays only. Eligible patients were identified by the study nurse from among patients presenting to the triage nurse in the waiting room by asking, "Do you have a cough and/or difficulty breathing?". Those 15 years and older who responded positively were eligible for inclusion unless they refused to provide written consent, were known to have severe psychiatric problems or required urgent care. Enrolment took place during usual consulting hours (07 h 30 to 17 h 00 from Mondays to Fridays).

The diagnosis of suspected pulmonary tuberculosis was made on-site independently by a nurse using the guideline without radiology, primary care physicians with the assistance of a chest radiograph and later, off-site by a respiratory physician also with the assistance of chest radiography.

### Nurse practitioner diagnosis of suspected tuberculosis

The nurse practitioner obtained a medical history from each patient, recorded vital signs and made up to three diagnoses in each subject based upon the PALSA guideline. As the purpose of the study was to assess the performance of the guideline, the nurse was instructed to follow the guideline's diagnostic algorithms rigidly and not to employ additional judgement. She remained blinded to the results of the radiographs and the assessments made by the primary care and respiratory physicians.

### Primary care physician diagnosis of tuberculosis

On completing the interview with the nurse, patients were assessed by one of two primary care physicians who were blinded to the nurse using the guideline's diagnoses. Both primary care physicians had over 6 years postgraduate clinical experience. They performed flow volume loops before and after inhalation of 200 μg of salbutamol administered from a pressurised metered dose inhaler, using a PM1 Spirometer (Erich Jaeger^®^, Germany). A chest radiograph (lateral and postero-anterior views) was performed on each patient. The primary care physicians systematically recorded the patients' clinical details, the results of spirometry and chest radiography, and their diagnoses made without the assistance of the PALSA guideline. They recorded their levels of confidence in each diagnosis made, using a 5-point Likert scale (1 representing the lowest level of confidence and 5 the highest level of confidence or certainty). In all subjects in whom even a low level of suspicion of tuberculosis was recorded, sputum specimens for examination and culture were obtained. A first specimen was collected immediately and a second specimen the following day. The sodium citrate method was used to process the smears, which was examined for Acid Fast Bacilli (AFB) by one reader after staining with Ziehl-Neelsen. A positive smear was reported if at least one AFB per 100 immersion fields. Sputa were cultured on a slope of Löwenstein-Jensen medium and incubated at 37°C for about six weeks. Bacteriological confirmation of tuberculosis was accepted if two sputum smears collected on different days were positive as reported above, or the presence of a positive culture and one or no positive smear.

### Respiratory physician diagnosis of suspected tuberculosis

All records and investigations collected by the primary care physicians, were provided off-site to one of two specialist respiratory physicians, who were required to make clinical diagnoses, including suspected TB. They were blinded to the nurse using the guideline and primary care physicians' diagnoses, to whether sputum had been tested, and to the results of sputum examination. The respiratory physicians recorded up to four diagnoses on each patient, and recorded the certainty of each diagnosis in the same manner as the primary care physician (see above).

### Statistical analysis

The primary endpoint was performance of the nurse using the guideline as the first part of a two-step screening process. The respiratory physician diagnosis served as the comparator for the identification of patients requiring such screening. The sensitivity, specificity, positive and negative predictive values, positive and negative likelihood ratios, and areas under receiver operating characteristic curves were calculated for each test compared with reference standards. Ninety-five percent confidence intervals (95% CI) for proportions were calculated based on binomial distributions.

Logistic regression models identified clinical features that independently predicted diagnoses of suspected and bacteriologically-proven tuberculosis. Potential predictors were selected for inclusion in logistic regression models (after examining cross tabulations between each predictor and the reference standard diagnosis) if significantly (P < 0.05) associated with the outcome and if the crude odds ratios were more than 2 or less than 0.5. Variables were excluded from the models if not independently associated with the reference standard, and if their odds ratios were less than 0.5 and more than 2, aiming for a parsimonious model of powerful predictors. Independent (mutually adjusted) associations between each predictor and the reference standard diagnosis were then expressed as likelihood ratios instead of odds ratios. This entailed adjusting crude likelihood ratios with shrinkage factors obtained by logistic regression, as described by Spiegelhalter and Knill-Jones [14]. The adjusted likelihood ratios of different predictors can be multiplied by each other because they are independent. Confidence intervals for adjusted likelihood ratios were estimated by non-parametric bootstrapping of logistic regressions, with 2000 replications, and using the bias-corrected percentile method [15]. Data were analysed using STATA release 8 statistical software.

The study was approved by the research ethics committee of the University of Cape Town. Written informed consent was obtained from all patients before participation.

## Results

### Patients' characteristics

**Figure 1:**
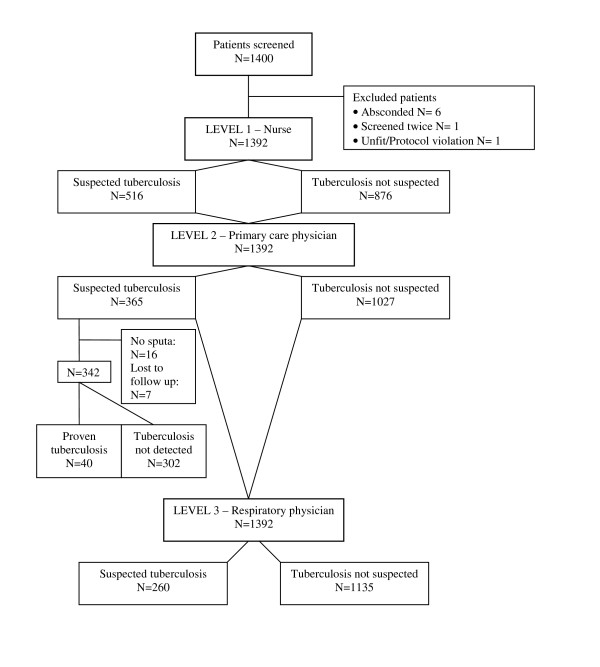
Flow chart of patients with diagnoses of suspected tuberculosis as made by nurse, primary care physician and respiratory physician, and proven tuberculosis as made by the primary care physician.

Fourteen hundred patients were enrolled and 1392 were eligible for analysis (Figure 1). Eight were excluded; 6 withdrew consent, 1 was screened twice and 1 was unable to give a detailed history. The median age of those analysed was 48 years (range 15–89) and 63% were female. Most patients were current smokers (48%) or ex-smokers (29%), and 63% (873) had previously been diagnosed with obstructive lung disease (asthma in 592 (43%) and COPD in 241 (20%)), while 38% had a history of cardiovascular disease (including hypertension, ischaemic and other forms of cardiac disease). Two hundred and two (15%) reported previously having been treated for tuberculosis (Table 1).

### Nurse versus primary care physician

The nurse diagnosed 516 (37%) patients as TB suspects, whereas the primary care physicians identified 365 (26%) of patients as suspected TB. The diagnostic accuracy of the nurse using the guideline as compared to the primary care physicians' diagnoses (Table 2) was: sensitivity 76% (95%CI 71%–79%), specificity 77% (95%CI 74%–79%), positive predictive value (PPV) 53% (95%CI 49%–58%), and negative predictive value (NPV) 90 (95%CI 88%–92%).

Sputum for microbiological testing was obtained from 342 (94%) of the primary care physicians TB suspects. Sputum could not be obtained in 23 (6%) patients. Tuberculosis was confirmed bacteriologically in 40 (12%) of the 342 patients investigated by sputum examination. Twenty four (60%) were smear positive, 5 (12%) smear and culture positive, and 11 (28%) culture positive only.

The sensitivity of the nurse using the guideline to detect patients with proven tuberculosis was 90% (95%CI 76%–97%), specificity was 65% (95%CI 63%–68%), PPV was 7% (95%CI 5%–10%), and NPV was 99.5% (95%CI 98.8%–99.8%) (Table 2). Eight (20%) of the 40 patients had reported cough and/or breathing for less than two weeks and 16 (40%) were also diagnosed with asthma or COPD by the primary care physicians. The proportion of patients with bacteriological confirmation increased with increasing certainty of the primary care physicians diagnostic rating (Table 3).

### Nurse versus respiratory physician

The respiratory physicians identified 262 (19%) patients as suspected tuberculosis. The primary care physicians identified 152 more patients for TB screening than the respiratory physicians, 6 of whom were later proven to have tuberculosis. The diagnostic accuracy of the nurse using the guideline compared to the respiratory physicians' diagnoses of suspected TB was as follows (Table 2): sensitivity 73% ((95%CI 67%–78%), specificity 71% (95%CI 68%–74%), PPV 37% (33%–41%), NPV 92% (95%CI 90%–94%), and area under the ROC curve 0.72 (95%CI 0.69–0.75).

### Symptoms predictive of tuberculosis

The most common symptoms in patients proven to have TB were cough (100%), followed by difficult breathing (70%), new sputum production (63%), loss of weight (50%), and night sweats (50%) (Table 4). Three clinical features independently predicted the diagnosis of TB. In decreasing order they were pleuritic chest pain, weight loss, night sweats (Table 5). The presence of all 3 increased the likelihood of TB 17 times, compared to if none were present. Features associated with the diagnosis of suspected TB by the respiratory physicians were haemoptysis, weight loss, night sweats, previous TB, pleuritic chest pain, absence of difficulty breathing, and absence of wheeze. The presence of all 7 increased the likelihood of the respiratory physicians diagnosing TB 594-fold.

## Discussion

In primary care in many developing countries, the initial responsibility for identifying patients who require screening for tuberculosis lies with nurse practitioners, especially in rural clinics. The PALSA guideline, which integrates symptoms and signs predicting tuberculosis with those of other respiratory diseases, serves as a diagnostic aid and as a potential means of increasing TB case detection, particularly in settings where the resources, skills and knowledge base is limited. The current study confirms that a nurse practitioner using the guideline is able to perform this task with sufficient accuracy when compared with the alternative – a primary care physician with access to more clinical details, and chest radiography.

The diagnostic utility of the PALSA guideline as demonstrated in this study is supported by the results of a pragmatic randomised control trial in which the guideline was used in rural clinics in the Free State province of South Africa [16]. In this study, use of the guideline, supported by illustrated materials and an educational outreach programme was associated with a 68% increase in the rate of tuberculosis case detection.

The fact that the nurse using the guideline demonstrated a high sensitivity, negative predictive value, and positive likelihood ratio when compared to the respiratory and primary care physicians, validates the approach of using syndromic 'diagnoses' in primary care for identifying patients requiring screening for tuberculosis. In such settings, high sensitivity is achieved at the expense of specificity. Thus, while it is unlikely that a 'positive' case will be missed, the low specificity will result in additional effort and expenditure on false positive patients. In the case of tuberculosis this will result in additional use of resources for sputum examination. This is reflected in the ratio of suspected to bacteriologically-confirmed cases which for the nurse using the guideline was 13:1, compared with 9:1 and 5:1 for the primary care and respiratory physicians, respectively. These values compare satisfactorily with the ratio for high prevalence populations of 10:1 recommended for high prevalence countries [17].

While the performance of the PALSA guideline was satisfactory in the population in which it was tested, it is recognised that the efficiency of a screening test is influenced by disease prevalence, which in this study was 3%. If the tuberculosis prevalence had been higher, the yield from screening would have been higher. Conversely, in lower tuberculosis prevalence settings the yield from screening would be lower. The current guideline therefore appears appropriate for primary care conditions in South Africa where notification rates for bacteriologically-confirmed pulmonary TB was 612 per 100,000 in 2002 [18].

One of the challenges of diagnosing TB in primary care is distinguishing symptoms of TB from those of other common respiratory diseases [19]. The PALSA guideline encourages the nurse practitioner to consider more than one diagnosis, with the aim of not only improving TB case detection, but also of improving recognition and the care of patients with other respiratory diseases. A striking feature of our study is the high proportion of our study population (63%) who had previously been diagnosed with chronic airways disease (asthma or COPD), one of which was present in 30% of patients in whom TB was confirmed. This has been observed in other countries in which integrated diagnostic guidelines have been employed, and illustrates the need for integrated diagnostic guidelines that do not focus on TB alone, but seek to identify other common respiratory diseases that require management.

In common with other studies, cough, difficult breathing, sputum production, weight loss, and night sweats, were common presenting features in patients with bacteriologically-confirmed tuberculosis [10,21,22]. We also analysed the predictive value of individual symptoms in patients diagnosed and suspected of TB. The absence of difficulty breathing best predicted suspected tuberculosis (Table 4) and suggests that this symptom should not be included in diagnostic algorithms for TB. The independent predictors: night sweats, loss of weight, and pleuritic chest pain all formed part of the PALSA algorithmic definition of suspected tuberculosis. The guideline can be simplified further by removal of criteria that do not add to the prediction of suspected tuberculosis. The likelihood ratios (LRs) in our study have been adjusted for their non-dependence. They may be applied to individual patients by multiplication of the LRs of symptoms present in that patient. For example, a primary care physician was 53 times more likely diagnose tuberculosis if weight loss, pleuritic pain, and raised temperature were present and the patient was not producing sputum, than if none of these predictors was present (Table 4). The LRs, rather than the sensitivity and specificity, were derived to permit extrapolation of the results to other settings with different prevalences of disease [23].

Use of a two-week rather than a three week cut-off for TB suspects in a general primary care clinic setting in a high prevalence country has previously been shown to increase the case-detection rate [24]. In high prevalence areas a minority of patients with TB present with cough for less than 2 or 3 weeks. In our study restricting screening to those with a cough for 2 weeks or longer would have resulted in failure to detect one-fifth of the confirmed cases. In an Ethiopian study in a high TB and HIV burden areas, patients with a cough of short duration (of 1 to 3 weeks), when associated one or more of the following; weight loss, absence of response to a short course of antibiotics, and/or living in overcrowded place, were associated with the diagnosis of TB [25]. Thirty-five percent of such patients had TB. Our study confirms that in high TB prevalence settings, TB should be suspected in all patients with cough, even when of a shorter duration than 2 weeks.

The methodological strengths of our study are the large sample of clinically diverse patients, the prospectively recorded and detailed clinical information on each patient, and the blinded diagnoses made by the nurse and the non-specialist and specialist physicians. The sample of patients was representative of those attending a primary health care clinic with respiratory symptoms. The fact that tuberculosis was diagnosed in patients presenting with other diagnoses emphasises the importance of considering tuberculosis in all patients at each visit in high prevalence areas even when other diagnoses are known. The study's main limitation was the absence of sputum testing in patients in whom the primary care physicians did not suspect tuberculosis. We were therefore not able to use bacteriological diagnosis of TB as the reference standard. However, our aim was to examine the performance of the first stage of a 2-stage screening process; the nurse using a guideline against the generally accepted approach of a primary care physician who has access to sputum testing and a chest radiograph. The availability of chest radiography may be useful in detecting in TB with typical chest radiograph findings, [26] and would have therefore been likely to have improved the diagnostic accuracy of the primary care physicians. This is supported by the association of the certainty of diagnosis of the primary care physicians with sputum positivity, suggesting that it is unlikely that those that they viewed as not requiring sputum examination had active TB.

## Conclusion

The study shows that, compared with primary care physicians who had with access to chest radiographs and more clinical information, a nurse practioner using the PALSA syndromic respiratory guideline was able to identify patients who require bacteriological screening for pulmonary tuberculosis, from among those presenting with respiratory symptoms in a high tuberculosis prevalence area.

## Competing interests

The author(s) declare that they have no competing interests.

## Authors' contributions

RGE contributed to the initial concept, design, data collection, co-ordination and writing of the manuscript. EDB, MFZ, MOB, RS, SEO and CL contributed to the initial concept and design. MOB and RGE analysed the data. MOB, RGE and EDB contributed to the interpretation of data. RGE, EDB, MFZ contributed to the development of the first main draft of the guideline. LRF, AB, BM, and MOB to the revision of the guideline. MOB, BM, RGE, EDB, MFZ facilitated the guideline revision focus group discussions. MOB, EDB provided input into the preparation of the manuscript. All authors commented on the manuscript and gave approval for final submission.

## Pre-publication history

The pre-publication history for this paper can be accessed here:



## Supplementary Material

Additional file 1 Practical Approach to Lung Health in South Africa guideline. The guideline evaluated in this study. Click here for file

## Figures and Tables

**Table 1:** Patients' characteristics and respiratory physicians' diagnoses

**Characteristics**	**Median (range)**
Age (range), years	48 (15–89)
	**No. (%) (N = 1392)**
Female, No. (%)	876 (63)
**Previously diagnosed respiratory illnesses, **	
Previous tuberculosis	202 (15)
On tuberculosis treatment**	2 (0.1)
Asthma	592 (43)
Chronic obstructive respiratory disease (COPD)	281 (20)
**Other current illnesses**	
Cardiovascular disease	523 (38)
Diabetes	107 (8)
Allergic rhinitis	225(16)
Smoking history*	
Never smoked	330 (24)
Ex-smoker	407 (29)
Current smokers	653 (47)
	
**Respiratory diagnoses made by specialist physicians†**	
Suspected tuberculosis	260 (19%)
Asthma	508 (36%)
Chronic obstructive pulmonary disease	381 (27%)
Acute exacerbation of asthma/COPD	293 (21%)
Lower respiratory tract infection	283 (20%)
Allergic rhinitis	245 (17%)
Chronic bronchitis	66 (5%)
Upper respiratory tract infection	40 (3%)

* Missing data for two patients ** Excluded from subsequent analyses

† Up to four diagnoses per patient

**Table 2:** Diagnostic accuracy of nurse practitioner diagnosis of suspected tuberculosis, compared to primary care physician diagnoses of suspected tuberculosis, and tuberculosis

	Tuberculosis suspected by the primary care physician	Tuberculosis diagnosed by the primary care physician
True positives	276	36
False positives	240	462
False negatives	89	4
True negatives	787	867
Sensitivity % (95%CI)	76 (71–79)	90 (76–97)
Specificity % (95%CI)	77 (74–79)	65 (63–68)
Positive predictive value % (95%CI)	53 (49–58)	7 (5–10)
Negative predictive value % (95%CI)	90 (88–92)	99.5 (98.8–99.8)
Likelihood ratio for positive test	3.2	2.6
Likelihood ratio for negative test	0.31	0.15
Area under the ROC curve (95%CI)	0.76 (0.74–0.79)	0.78 (0.73–0.82)

**Table 3:** Increasing probability of bacteriological confirmation of tuberculosis as primary care physicians' strength of suspicion increased.

Strength of suspicion	Positive predictive value n/N (%)
1 (weakest suspicion)	1/29 (3)
2	3/76 (4)
3	6/85 (7)
4	7/65 (11)
5 (strongest suspicion)	23/87 (26)
All tuberculosis suspects	40/342 (12)

Non-parametric test for trend: P < 0.01

**Table 4:** Prevalence of symptoms among patients diagnosed with TB

Symptom	Proven TB (n = 40)
Cough	40 (100)
Difficult breathing	28 (70)
New sputum production	25 (63)
Current smoker	21 (53)
Loss of weight	20 (50)
Night sweats	20 (50)
Male	20 (50)
Pleuritic chest pain	11 (30)
Previous TB	12 (30)
No tight chest	10 (25)
No wheeze	8 (20)
Ex-smoker	7 (18)
Increased sputum production	6 (15)
Haemoptysis	5 (13)
Sputum colour change	5 (13)
Fever	2 (5)

**Table 5:** Independent and combined likelihood ratios for predictors of suspected or diagnosed tuberculosis: logistic regression models

Reference standard	Predictors	Positive likelihood ratio	(95% CI)	Negative likelihood ratio	(95% CI)
Tuberculosis suspected by respiratory physician*					
	Previous tuberculosis	2.6	(1.9–3.5)	0.75	(0.67–0.84)
	Haemoptysis	4.8	(2.2–10.3)	0.91	(0.86–0.96)
	Night sweating	2.8	(1.8–4.2)	0.77	(0.68–0.87)
	Weight loss	3.5	(2.3–5.6)	0.75	(0.66–0.84)
	Pleuritic pain	2.5	(1.5–4.1)	0.90	(0.84–0.95)
	No difficulty breathing	1.6	(1.2–2.1)	0.88	(0.80–0.96)
	No wheeze	1.2	(1.1–1.4)	0.70	(0.53–0.92)
	All 7 predictors	594	(232–1821)	0.22	(0.17–0.29)
Tuberculosis diagnosed by primary care physician**					
	Weight loss	2.9	(1.3–5.8)	0.81	(0.67–0.96)
	Night sweats	2.0	(1.0–4.6)	0.76	(0.54–0.97)
	Pleuritic pain	2.9	(1.0–4.8)	0.82	(0.63–1.00)
	All 3 predictors	16.7	(5.9–29.4)	0.50	(0.36–0.65)

* area under ROC curve for model = 0.85. ** area under ROC curve for model = 0.74
