# Supplementary material for: Diagnostic accuracy of an integrated respiratory guideline in identifying patients with respiratory symptoms requiring screening for pulmonary tuberculosis: a cross-sectional study
Source: BMC Pulm Med. 2006 Aug 25;6:22. doi: 10.1186/1471-2466-6-22 (PMC1569870; doi:10.1186/1471-2466-6-22)
Supplement: Additional file 1 — Practical Approach to Lung Health in South Africa guideline. The guideline evaluated in this study. [file 1471-2466-6-22-S1.pdf]

# PRACTICAL APPROACH TO LUNG HEALTH IN SOUTH AFRICA (PALSA) GUIDELINES

## First-Level Primary Care Management of Respiratory Diseases

Approach to the adult patient who presents with difficult breathing and/or cough.

# CONTENTS


## PATIENT WITH DIFFICULT BREATHING AND/OR COUGH

Classify according to symptoms 1
Symptoms < 2 weeks: ASSESSMENT AND INITIAL MANAGEMENT 2
Further treatment of the wheezing patient: ASTHMA/COPD EXACERBATION 3
Discharge plan for the wheezing patient who has responded to treatment 4
Further treatment of the patient with fever and/or pain on breathing and coughing: LOWER RESPIRATORY TRACT INFECTION 5

## UPPER RESPIRATORY TRACT INFECTIONS

Mildly ill patient with runny/blocked nose: RHINITIS 6
Mildly ill patient with pain and/or tenderness over sinuses: ACUTE SINUSITIS 7
Mildly ill patient with sore throat: ACUTE PHARYNGITIS, TONSILLITIS, ORAL CANDIDA 8
Mildly ill patient with ear problem: ACUTE AND CHRONIC EAR PROBLEMS 10
Dry mopping the ear 11

## SYMPTOMS ≥ 2 WEEKS

Diagnosing obstructive lung disease 12
Management of chronic asthma 13
Management of chronic obstructive pulmonary disease (COPD) 14
Chronic cough with or without sputum production; no breathlessness: CHRONIC BRONCHITIS 15

## TUBERCULOSIS (TB)

Diagnosing TB 16
Sputum results 17
Follow-up plan for Regimen One 18
Follow-up plan for Regimen Two 19

## HIV/AIDS

Suspecting HIV/AIDS 20
Follow-up of the known HIV-positive patient 21
Who is eligible for long-term cotrimoxazole (Bactrim) prophylaxis? 22

# CLASSIFY ACCORDING TO SYMPTOMS

- **Cough AND/OR**
- **Difficult breathing (defined as breathlessness at rest or on activity, wheeze and/or tight chest)**

## ASK ABOUT, AND RECORD

- Name
- Age
- Medical history
- Presenting symptoms
- Purpose of the visit

**If the purpose of this visit is to treat and assess:**

- Worsening of symptoms or
- New symptoms

**If unsure of diagnosis**

**Symptoms present < 2 weeks**

Go to page 2

**Symptoms present ≥ 2 weeks**

**Cough with or without sputum production**

**Exclude TB**
(Go to page 16)
Consider **Chronic Bronchitis**
(Go to page 15)

**Cough and difficult breathing**

**Difficult breathing alone**

**Exclude TB**
(Go to page 16)
Consider **Asthma or COPD**
(Go to page 12)

**If continued treatment of known lung disease with:**

- No worsening of symptoms
- No new symptoms
- No uncertainty about diagnosis

- **Asthma** (Go to page 13)
- **COPD** (Go to page 14)
- **TB** (Go to page 16)
- **HIV/AIDS** (Go to page 20)

# SYMPTOMS < 2 WEEKS: ASSESSMENT AND INITIAL MANAGEMENT

## IF ONE OR MORE SYMPTOMS PRESENT, ASSESS SEVERITY

| | SEVERE | MILD | NORMAL |
|---|---|---|---|
| **BREATHLESSNESS** | At rest or while talking | While walking | Normal |
| **MENTAL STATE** | May be agitated or confused | | Normal |
| **USE OF BREATHING MUSCLES** | Prominent | May be normal | Normal |
| **BREATH RATE** | ≥ 30 per minute | 20 - 29 per minute | < 20 per minute |
| **HEART RATE** | ≥ 120 per minute | 100-119 per minute | < 100 per minute |
| **HAEMOPTYSIS** | ≥ Tablespoon of frank blood | Blood streaking | Normal |

### INITIAL MANAGEMENT OF SEVERE PATIENTS

Airway: Position for greatest ease of breathing.
Breathing: 40% Face-mask oxygen or at 4 L/min via nasal prongs.
Call ambulance.
Doctor: Phone or refer.
Extra emergency treatment:

**Wheezing or tight chest**

**Temperature ≥ 38° C**

### SEVERE ACUTE ASTHMA/COPD EXACERBATIONS

- 4-8 puffs beta-agonist via spacer every 20 minutes in the first hour, then hourly depending on response
  OR
  Nebulise beta-agonist every 20 minutes, then hourly depending on response
- Oral prednisone 40mg

### SEVERE LOWER RESPIRATORY TRACT INFECTION

Give: Amoxycillin 1 gm orally or if penicillin-allergic Erythromycin 500 mg orally

### ASK, LISTEN:

Wheezing, tight chest?

Most likely **asthma** or **chronic obstructive airways disease (COPD) exacerbation.**

Go to page 3

### ASK, MEASURE:

Fever and/or pain on breathing or coughing and/or sputum production

Most likely **LRTI, TB** or **suppurative lung disease.**

Go to page 5

### ASK, LOOK:

- Runny nose
- Sore throat
- Pain and/or tenderness over sinuses
- Ear problem

**UPPER RESPIRATORY TRACT INFECTION**

Go to page 6-11

# FURTHER TREATMENT OF THE WHEEZING PATIENT: ACUTE ASTHMA/COPD EXACERBATION

- 4 puffs beta-agonist via spacer every 20 minutes for one hour then reassess.

  OR

  Nebulise using beta-agonist every 20 minutes for one hour then reassess.
- Give 1 dose of oral prednisone 40 mg stat.

**REASSESS SYMPTOMS AFTER 1 HOUR**

**BETTER OR NO SYMPTOMS**

**OBSERVE FOR ONE MORE HOUR, THEN FOLLOW DISCHARGE PLAN ON PAGE 4**

**NO CHANGE**

**REPEAT ABOVE TREATMENT AND ASSESS WITHIN ONE HOUR**

If worsening of symptoms, treat as severe and refer.

If no response within two hours, refer.

**WORSE**

**FOLLOW TREATMENT PLAN FOR SEVERE PATIENT ON PAGE 2.**

# DISCHARGE PLAN FOR THE WHEEZING PATIENT WHO HAS RESPONDED TO TREATMENT

•Increase the dose and frequency of the inhaled bronchodilator to a maximum of 2 puffs 4 times a day.

•If the patient is already on inhaled corticosteroids: check compliance (are medications taken twice a day, every day)
: check inhaler technique (are the inhalers used correctly)

•If poor compliance and/or technique instruct patient on correct drug usage.

•Give 40mg of prednisone orally (once daily) for 7 days to patients with the following:

-History of recent emergency visits for asthma.

-Worsening of asthma symptoms in the months or weeks prior the onset of the acute attack.

-History of previous hospital or intensive care unit admission for asthma.

•If the patient reports a cough with new or increased sputum production and/or change in sputum colour (yellow, green) and/or fever, add Amoxycillin 500mg three times a day for 7 days OR if penicillin-allergic, Erythromycin 500mg four times a day for 7 days.

•If the underlying lung condition is unknown, go to page 12 to make diagnosis.

•Encourage all patients to stop smoking cigarettes, pipes or dagga.

•Book follow-up visit before medicines are expected to run out.

**TELL PATIENT TO RETURN IF:**

•Symptoms get worse.

•Not better after a course of oral prednisone has been completed.

# FURTHER TREATMENT OF THE PATIENT WITH FEVER AND/OR PAIN ON BREATHING OR COUGHING: LOWER RESPIRATORY TRACT INFECTION

## IS THIS PATIENT AT HIGH RISK OF SEVERE RESPIRATORY INFECTION?

- ≥ 60 years old
- Frail with suspected AIDS
- Known: Lung disease
  Heart disease
  Liver disease
  Diabetes Mellitus

Immediately give 1 gram Amoxycillin orally
OR
If penicillin-allergic, Erythromycin 500mg orally
AND
**REFER TO NEXT LEVEL FACILITY OR CLINIC DOCTOR**

## NOT AT HIGH RISK OF SEVERE RESPIRATORY INFECTION?

- Bed rest at home
- Encourage high fluid intake
- No smoking
- Treat pain and fever with paracetamol 1-2 tablets 4 times a day.
- If new or increased sputum production with colour change, prescribe Amoxycillin 500mg orally three times a day for 7 days OR if penicillin-allergic, Erythromycin 500mg orally 6 hourly for 7 days.
- Look for signs of HIV/AIDS (Go to page 20)
- **Ask about symptoms of TB** (such as loss of weight, night sweats) (Go to page 16)

**Refer if:**

- Getting worse, or no response.
- Still not completely better within 7 days.

# MILDLY ILL PATIENT WITH RUNNY/BLOCKED NOSE: RHINITIS

**Ask about associated**
- Mild sore throat
- Fever

Consider: **Common cold**

**REASSURE PATIENT THAT ANTIBIOTICS ARE NOT NECESSARY.**

Consider oxymetazoline 0.05% nose drops, 2 drops in each nostril every 6-8 hours for **no longer** than 1 day.

**If:**

Symptoms on most days for ≥ 4 weeks, ask about
- Sneezing
- Itching

Consider: **Allergic rhinitis (hayfever)**

**INTERMITTENT**
< 4 days per week

- 0.9% saline nose drops.
- Chlorpheniramine 4mg 3-4 times a day when necessary Beware: Side-effect is sedation.

**PERSISTENT**
≥ 4 days per week

- 0.9% saline nose drops.
- Chlorpheniramine 4mg 3-4 times a day when necessary Beware: Side-effect is sedation.
- Refer to next level facility for steroid nasal spray.

# MILDLY ILL PATIENT WITH PAIN AND/OR TENDERNESS OVER SINUSES: ACUTE SINUSITIS

- Clear nasal discharge.
- Mild pain over sinuses.
- Post-nasal drip.

Consider: **Viral sinusitis**

**REASSURE PATIENT THAT ANTIBIOTICS ARE NOT NECESSARY.**

- Instruct patient to mix 1/2 teaspoon salt + 1 teaspoon bicarbonate of soda in 500ml lukewarm water. Sniff up each nostril every 4-6 hours.
  OR
  0.9% Sodium chloride drops in each nostril every 4-6 hours.
- Oxymetazoline 0.05% nose drops, 2 drops in each nostril every 6-8 hours for **no longer** than 5 days.
- Paracetamol 1-2 tablets 4 times a day.

- Symptoms $\geq$ 7 days.
- Severe symptoms regardless of duration.
- Pussy nasal discharge.
- Face or tooth pain and tenderness.

Consider: **Bacterial sinusitis**

- Amoxycillin 500mg orally three times a day for 10 days
  OR
  If penicillin-allergic, give cotrimoxazole (Bactrim) 2 tablets (80/400mg) twice a day for 5 days.
- Instruct patient to mix 1/2 teaspoon salt + 1 teaspoon bicarbonate of soda in 500ml lukewarm water. Sniff up each nostril every 4-6 hours.
  OR
  0.9% Sodium chloride drops in each nostril every 4-6 hours.
- Oxymetazoline 0.05% nose drops, 2 drops in each nostril every 6-8 hours for **no longer** than 5 days.
- Paracetamol 1-2 tablets 4 times a day.

**Refer if:**
- Tooth abscess suspected.
- Swelling around eye or face.
- Failure to respond to medication after 10 days.

# MILDLY ILL PATIENT WITH SORE THROAT:
# ACUTE PHARYNGITIS, TONSILLITIS, ORAL CANDIDA

## RED THROAT WITHOUT PUS

Consider: **Pharyngitis**

**REASSURE PATIENT THAT ANTIBIOTICS ARE NOT NECESSARY.**

- Salt water mouthwash (1/2 teaspoon salt in a glass of warm water). Gargle twice a day.
- Paracetamol 1-2 tablets 4 times a day.

## RED THROAT, WITH PUS OR WHITE PATCHES ON TONSILS

Consider: **Bacterial tonsillitis**

- Salt water mouthwash (1/2 teaspoon salt in a glass of warm water). Gargle twice a day.
- Phenoxymethylpenicillin (Pen VK) 500mg orally every 6 hours for 10 days.
  OR
  If penicillin-allergic, give Erythromycin 250mg 6 hourly before meals for 10 days.
- Paracetamol 1-2 tablets 4 times a day.

**Refer if:**
- Severe swallowing problems.
- Inability to open mouth.
- More than 4 documented episodes per year.

## WHITE PATCHES ON CHEEKS, GUMS, TONGUE AND PALATE

Consider: **Oral candida (thrush)**

- Nystatin lozenges 100 000 IU - 4 times a day for 10 days.
  OR
  Nystatin 100 000 IU/ml 1-2 ml 4 times a day for 10 days.
- Exclude HIV infection.
  (Go to page 20)

**Refer if:**
- No response to Nystatin within 5 days. Fluconazole to be prescribed by doctor.
- Extensive disease.
- Recurrent episodes.

## Examine the cheeks

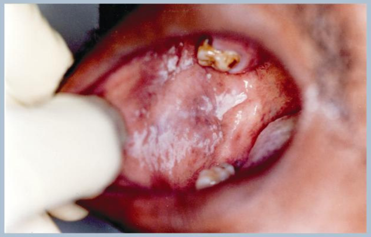

*Candida*

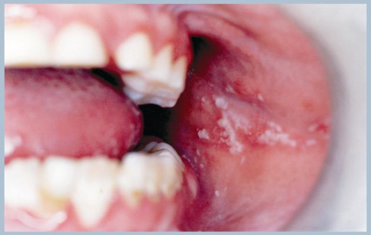

*Candida*

## Examine the tongue

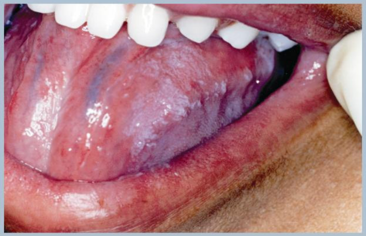

*Candida*

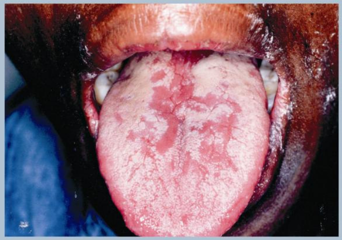

*Candida*

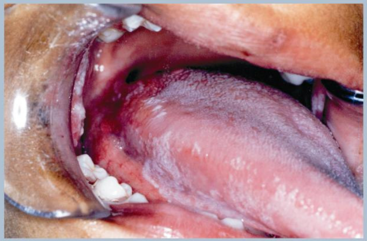

*Candida*

## Examine the palate

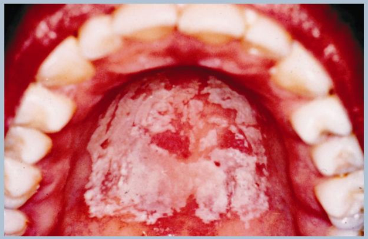

*Candida*

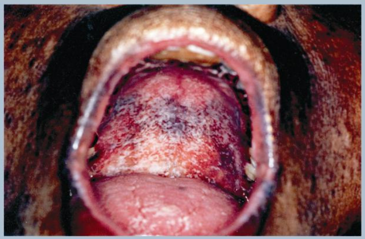

*Candida*

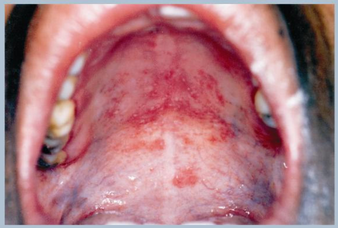

*Candida*

# MILDLY ILL PATIENT WITH EAR PROBLEM: ACUTE AND CHRONIC EAR PROBLEMS

## LOOK IN EAR

### OUTER EAR AND EAR CANAL

#### OTITIS EXTERNA

**Inflammation or infection of skin of the outer ear**

- Give Flucloxacillin 250mg orally 4 times a day
  OR
  If penicillin-allergic, give Erythromycin stearate 250mg 4 times a day for 5 days.
- Paracetamol 1 - 2 tablets 4 times a day for pain.

**Inflammation or infection of ear canal**

- Give Flucloxacillin 250mg orally 4 times a day.
  OR
  If penicillin-allergic, give Erythromycin stearate 250mg 4 times a day for 5 days
- 2% Acetic acid in alcohol, 6 hourly for 5 days.
- Dry mop ear.
  (Go to page 11)
- Paracetamol 1 - 2 tablets 4 times a day for pain.

**Refer if:**
- Diabetic.
- If no response within 48 hrs.

### EARDRUM

#### OTITIS MEDIA

#### ACUTE

**Inflamed or bulging eardrum, or pus from ear for < 2 weeks**

- Dry mop ear.
  (Go to page 11)
- Amoxycillin 250mg orally three times a day for 5 days.
  OR
  If penicillin-allergic, give Bactrim 2 tablets (80/400mg) twice a day for 5 days.
- Paracetamol 1-2 tablets 4 times a day for pain.

**Refer if:**
- No response after 5 days.
- Swelling and tenderness of skin behind ear.
- Persistent pain.

#### CHRONIC

**Perforation and pus from ear for ≥ 2 weeks**

- The ear can only heal if dry.
- Dry mop ear.
  (Go to page 11)
- Paracetamol 1-2 tablets 4 times a day for pain.

**Refer if:**
- No improvement after 4 weeks.
- Foul-smelling discharge.
- Large hole in eardrum.
- Hearing loss.
- Pain.

## Otitis Externa

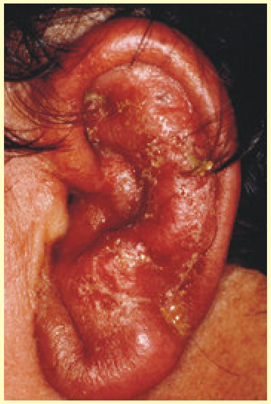

*Inflamed, swollen outer ear*

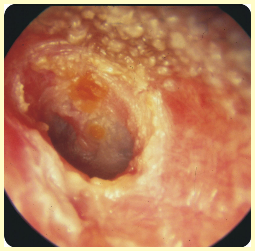

*Red swollen ear canal*

## Acute Otitis Media

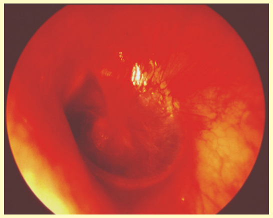

*Inflamed eardrum*

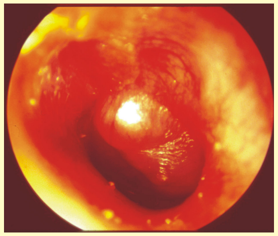

*Bulging eardrum*

## Chronic Otitis Media

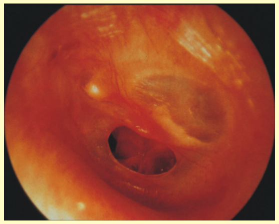

*Dry perforation*

# DRY MOPPING THE EAR

**Demonstrate method to patient.**

- Roll a piece of paper towel into a wick.
- Insert wick into ear and remove once it is wet.
- Repeat 4 times a day until ear is dry.
- Insert acetic acid ear drops if indicated (go to page 10) - 4 drops in affected ear.
- Never leave the wick or any other object inside the ear.

# DIAGNOSING OBSTRUCTIVE LUNG DISEASE

**It is not always easy to decide whether a patient has asthma or COPD as the symptoms may be similar, or both diseases may be present.**
**A few questions may help with the diagnosis.**

## Ask if:

- Symptoms started during childhood or early adulthood.
- History of hayfever, eczema and/or allergies.
- Family history of asthma.
- Symptoms only during attacks with periods of normal breathing in between.
- Symptoms are usually worse: at night; in the early hours of the morning; during an upper respiratory tract infection or when the weather changes.
- Symptoms improve or disappear after using inhaler.

**TREAT AS ASTHMA.**
**REFER TO DOCTOR WITHIN 1 MONTH**
**Go to page 13**

## Ask if:

- Symptoms started later in life (usually after the age of 35 years).
- Symptoms slowly worsened over a long period of time.
- Long history of daily or frequent cough and sputum production (usually starts long before the onset of shortness of breath).
- Short of breath for most of the day, rather than at night or during the early hours of the morning only.
- History of heavy smoking eg. more than 20 cigarettes / day for 15 years or more.

**TREAT AS COPD.**
**REFER TO DOCTOR WITHIN 1 MONTH.**
**Go to page 14**

**(If unsure, treat as asthma)**

If $\leq$ 1 feature of asthma, and no significant history of smoking, consider a cardiac or non-lung cause of breathlessness, especially if associated hypertension, ischaemic heart disease and/or diabetes mellitus.

# MANAGEMENT OF CHRONIC ASTHMA

**The aim of asthma management is to obtain complete control of all features of asthma.**

Aim for:

1) Minimal (ideally no) daytime and night time symptoms
2) Minimal or no exacerbations (asthma attacks)
3) Minimal need for quick-relief medications
4) No limitations of daily activities

## ASSESS CONTROL OF ASTHMA BY ASKING ABOUT DAY AND NIGHT TIME SYMPTOMS

| LEVEL OF CONTROL | WELL-CONTROLLED | MODERATE CONTROL | POOR CONTROL |
|---|---|---|---|
| **Daytime symptoms per week** | <2 times / week | 2-4 times / week | Continuous |
| **Night time symptoms per month** | <2 times / month | 2-4 times /month | Frequent |

| LEVELS OF TREATMENT | LOW (if well-controlled) | MODERATE (if moderate control) | MAXIMUM (if poor control) |
|---|---|---|---|
| Inhaled salbutamol | 2 puffs when needed | 2 puffs when needed | 2 puffs when needed<br>May be required 4-6 times per day. |
| Inhaled corticosteroids | 200-400 micrograms / day | 800 micrograms / day | 800-1600 micrograms / day |
| Slow-release theophylline<br>Doctor to initiate | - | - | 1 tablet twice a day |
| Oral prednisone | - | - | 40mg orally (once daily) for 14 days to gain rapid control. |

**REVIEW EVERY 3 MONTHS**

### IF COMPLETE CONTROL AT ANY LEVEL OF TREATMENT

- Continue current medication.
- At next visit, reduce treatment to previous level (step-down) if control is still complete.
- Schedule next appointment.

### IF POOR CONTROL AT ANY LEVEL OF TREATMENT

- Increase to next level of treatment (step-up).
- Consider adding prednisone 40mg orally once daily for 7 days and reassess in 1 month.

**Refer if poor control despite stepping-up.**

# MANAGEMENT OF CHRONIC OBSTRUCTIVE PULMONARY DISEASE (COPD)

**The aim of COPD management is to:**

- Encourage patients to stop smoking in order to prevent worsening of disease.
- Improve symptoms with inhaled bronchodilators.
- Recognise and treat acute exacerbations early.

**ENCOURAGE THE PATIENT TO STOP SMOKING**

**Ask**: Identify and document all tobacco use at each visit.
**Advise**: Strongly urge the patient to quit.
**Assess**: Determine willingness to make a quit attempt.
**Assist**: Help the patient to quit.
**Arrange**: Schedule follow-up contact.

| | MODERATE | SEVERE | SEVERE COPD WITH COMPLICATIONS | INFECTION |
|---|---|---|---|---|
| **Symptoms** | Mild breathlessness on usual activity | Breathlessness on minimal activity or continuously. | Ankle oedema | Increased sputum purulence or colour change to yellow/green |
| **Treatment Options**<br>**Bronchodilators**<br>**Inhaled salbutamol** | 2 puffs when needed | 2 puffs when needed | 2 puffs 4 times a day | 2 puffs when needed |
| **Inhaled ipratopium bromide** | - | 2 puffs when needed (up to 4 times per day) | 2 puffs 4 times a day | 2 puffs when needed (up to 4 times per day) |
| **Theophylline** | 1 tablet 2 times per day | 1 tablet 2 times per day | 1 tablet 2 times per day | 1 tablet 2 times per day |
| | | | **Refer for diuretics if ankle oedema** | Amoxycillin 500mg three times a day for 7 days<br>OR<br>If penicillin-allergic, Erythromycin 500mg four times for 7 days.<br>Prednisone 40mg orally (once daily) for 14 days |

**REVIEW EVERY 3 TO 6 MONTHS**

# CHRONIC COUGH WITH OR WITHOUT SPUTUM PRODUCTION; NO BREATHLESSNESS: CHRONIC BRONCHITIS

- **Usually in heavy smokers, or those with lung damage.**
- Daily cough with or without sputum production for months or years.
- Usually begins in middle or old age.
- Heavy occupational (dust, mines, industry) or domestic air pollution (indoor fires or gas stoves) exposure in some.

## Treatment

**THE MOST EFFECTIVE TREATMENT IS TO REMOVE THE CAUSE!**

- All patients should be advised to stop smoking.
- If possible, avoid domestic pollution, occupational exposure and substance abuse (eg. dagga).

**Refer:**

- If no history of smoking.

# DIAGNOSING TUBERCULOSIS (TB)*

**SUSPECT TB WHEN:**

- Patient reports cough for ≥ 2 weeks.
- Unintentional weight loss.
- Loss of appetite.
- Night sweats and fever.
- Blood-stained sputum.
- Known HIV-positive or AIDS patients.

**METHOD OF SPUTUM COLLECTION**

**Patient:**

- Must stand in a well-ventilated room or outside.
- Rinse mouth with water.
- Take a deep breath, and cough forcibly.

**Nurse:**

- Must not stand in front of patient during the procedure.
- Replace and secure the lid immediately.
- Wash hands after handling specimen.
- Place specimen in bag and store in fridge while awaiting collection.

**TB SUSPECTED**

| **NEW, OR PREVIOUSLY CONFIRMED TB TREATED FOR < 4 WEEKS** | **PREVIOUS TB TREATED FOR ≥ 4 WEEKS** |
|---|---|
| Test sputum: Label bottles before dispensing them to patients. | |
| **Day 1:** For Acid-Fast Bacilli (AFB's).<br><br>**Day 2:** Early morning sputum, at home, for AFB's. | **Day 1:** For Acid-Fast Bacilli (AFB's).<br><br>**Day 2: Two** early morning sputa, at home.<br>• 1 for AFB's<br>• 1 for culture and sensitivity testing. |

* According to the South African Tuberculosis Control Practical Guidelines 2000

# SPUTUM RESULTS

**Sputum (AFB+AFB+)**

**Sputa (AFB+ AFB-)**
- Refer for CXR and schedule follow-up.

**CXR report suggests TB.**

**CXR report does not suggest active TB or other condition requiring immediate referral.**
- Repeat 1 sputum for AFBs
- Schedule follow-up

**Sputum AFB+**

**Sputum AFB-**
- Give Amoxycillin 250 mg orally 3 times a day for 7 days.
- Schedule follow-up.

**Little improvement**
- Repeat 1 sputum for AFBs

**Improvement**
- Suggests other respiratory diagnosis

**Sputum AFB+**

**Sputum AFB-**
- Refer to medical officer for CXR +\- culture

**Sputa (AFB-AFB-)**
- Give Amoxycillin 250mg orally 3 times a day for 7days.

**Little improvement**
- Repeat 1 sputum for AFBs

**Improvement**
- Suggests other respiratory diagnosis.

**Sputum AFB+**

**Sputum AFB-**
- Refer to medical officer for CXR +\- culture

## ACTIVE TB CONFIRMED

- Notify and register patient.
- If new case, or previous confirmed TB treatment for < 4 weeks, register as **NEW CASE**; **SPUTUM-POSITIVE PULMONARY TB** and start the intensive phase of **REGIMEN 1**. (Go to page 18)
- If previous TB treatment for ≥ 4 weeks, register as **RETREATMENT PATIENT**; **SPUTUM-POSITIVE PULMONARY TB**, and start the intensive phase of **REGIMEN 2.** (Go to page 19)
- Offer HIV test to all patients. (Go to page 20)
- Select DOT supervisor.

# INITIAL TREATMENT FOR REGIMEN ONE

| START INTENSIVE PHASE | < 50 kg | ≥ 50 kg |
|---|---|---|
| Rifampicin/Isoniazid/Pyrazinamide/Ethambutol 120/60/300/200mg (given 5 days a week). | 4 tablets | 5 tablets |

**At the end of 2 MONTHS of INTENSIVE treatment, take 2 sputa for AFBs. Schedule follow-up.**

**Sputa AFB- AFB-**

**Sputa AFB+ AFB- OR Sputa AFB+ AFB+**
- Continue intensive phase for 1 more month.

**At the end of 3 MONTHS, repeat 2 sputa for AFBs**
- Schedule follow-up.

**Sputa AFB- AFB-**

**Sputa AFB+ AFB- OR Sputa AFB+ AFB+'**
- Take sputum for culture and sensitivity.
- Schedule follow-up.
- If susceptible, continue. If resistant refer to MDR unit.

| START CONTINUATION PHASE | < 50 kg | ≥ 50 kg |
|---|---|---|
| Rifampicin/Isoniazid 150/100mg. | 3 tablets | - |
| Rifampicin/Isoniazid 300/150mg. | - | 2 tablets |

**At the end of 5 months of treatment, take 2 sputa for AFBs. Schedule follow-up.**

**Sputa AFB- AFB- or unable to produce sputum.**
- Stop treatment and register as **CURED.**
- Discharge from TB clinic.
- Refer HIV-positive patients to the general clinic for further management.

**Sputa AFB+ AFB- OR Sputa AFB+ AFB+**
- Register as **TREATMENT FAILURE.**
- Take sputum for culture and sensitivity
- Re-register as a **RETREATMENT** patient, and refer to follow-up plan for Regimen 2.

# TREATMENT PLAN FOR REGIMEN TWO

| START INTENSIVE PHASE | < 50 kg | ≥ 50 kg |
|---|---|---|
| Rifampicin/Isoniazid/Pyrazinamide/Ethambutol 120/60/300/200mg (given 5 days a week) PLUS | 4 tablets | 5 tablets |
| Streptomycin (given 5 days a week) intramuscularly. | 750mg | 1000mg |
| THIRD MONTH | | |
| Rifampicin/Isoniazid/Pyrazinamide/Ethambutol 120/60/300/200mg (given 5 days a week) ONLY | 4 tablets | 5 tablets |

**At 6 weeks review the susceptibility results of the initial sputum. If:**

**Susceptible**
- Continue treatment

**Resistant**
- Refer to MDR unit

**At the end of 3 months, repeat 2 sputa for AFBs**
- Schedule follow-up

**Sputa AFB- AFB-**

**Sputa AFB+ AFB- OR Sputa AFB+ AFB+**
- Repeat sputum for culture and sensitivity
- Schedule follow-up

- If susceptible, continue. If resistant refer to MDR unit.

| START CONTINUATION PHASE | < 50 kg | ≥ 50 kg |
|---|---|---|
| Rifampicin/Isoniazid 150/100mg + Ethambutol 400mg | 3 tablets + 2 tablets | - |
| Rifampicin/Isoniazid 300/150mg + Ethambutol 400mg | - | 2 tablets + 3 tablets |

**At the end of 7 months of treatment, take 2 sputa for AFBs. Schedule follow-up.**

**Sputa AFB- AFB- or unable to produce sputum.**
- Stop treatment and register as **CURED.**
- Discharge from TB clinic.
- Refer HIV-positive patients to the general clinic for management.

**Sputa AFB+ AFB- OR Sputa AFB+ AFB+**
- Register as **TREATMENT FAILURE.**
- Take sputum for culture and sensitivity.
- Refer to MDR unit.

# HIV/AIDS
# SUSPECT HIV/AIDS IN ALL WITH THE FOLLOWING:

- TB
- Recurrent respiratory infections
- Mouth lesions eg. Oral candida
- Skin infections eg. Herpes Zoster
- Severe weight loss
- Unexplained fever for > 4 weeks
- Sexually transmitted infections
- Painless swollen glands
- Long history of diarrhoea
- History of engaging in high-risk behaviour (eg. Vaginal, anal or oral sex without a condom)

## LOOK FOR

- White patches in the mouth, which are scratched off with difficulty, causing bleeding **(ORAL THRUSH/CANDIDA).**
- Painful rash with blisters, confined to one part of the body **(HERPES ZOSTER).**
- Bluish-black patches or lumps on skin or mouth **(KAPOSI'S SARCOMA)**.
- Evidence of severe loss of weight.
- Genital ulcers or discharge.

- **DO YOU SUSPECT HIV/AIDS ?**
- **DOES THE PATIENT REQUEST AN HIV TEST?**

## INFORM ABOUT VOLUNTARY CONFIDENTIAL COUNSELLING AND TESTING (VCCT)

Educate patient about HIV/AIDS, methods of transmission and risk factors.

Explain about VCCT:

Who will perform the counselling and the testing.
That it is completely voluntary.
That testing is confidential.
How testing is done.
When and how results are given.
What the results means.

- **If patient agrees to have VCCT, refer to the lay counsellor for testing.**
- **If a lay counsellor is not available, refer to health facility where testing is available.**

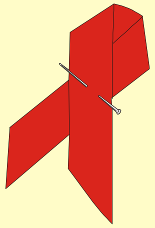

# FOLLOW-UP OF KNOWN HIV-POSITIVE PATIENT

## HIV POSITIVE

- Establish a relationship with the patient and encourage regular follow-up.
- Respect his/her right to confidentiality.
- Refer to the lay counsellor should the patient require further counselling.
- Encourage safer-sex practices.
- Provide medical care at each visit.
- Look for and treat HIV-related diseases.

### ORAL THRUSH/CANDIDA

Go to page 9

### ASYMMETRIC LARGE GLANDS

**Refer:**
For exclusion of extra-pulmonary TB.

### ANY OTHER HIV-RELATED DISEASES

**Refer to:**
South African Department of Health booklet: Recommendations for the prevention and treatment of opportunistic and HIV-related diseases in adult. (www.http://196.36.153.56/doh/aids/docs/adult.html)

## HIV NEGATIVE

- **Encourage safer sex practices.**

# WHO IS ELIGIBLE FOR LIFE-LONG COTRIMOXAZOLE (BACTRIM) PROPHYLAXIS? (2 SINGLE STRENGTH TABLETS (80/400MG) PER DAY)

- All HIV-infected TB patients .
- All symptomatic HIV patients (World Health Organisation (WHO) stage 2,3,4). Refer below.
- If previous diagnosis of Pneumocystis carinii pneumonia.
- Cotrimoxazole (Bactrim) prophylaxis is started at a higher-level facility.

## ADAPTED FROM THE WORLD HEALTH ORGANISATION (WHO) CLINICAL STAGING FOR HIV INFECTION

### STAGE 1

Without symptoms.
Acute viral illness following HIV infection.
Persistent swollen glands < 2 cm and symmetrical.

### STAGE 2

Unintentional weight loss.
Minor mouth and skin conditions (dry skin, mouth ulcers, fungal nail infections).
Herpes Zoster within the last 5 years.
Recurrent upper respiratory tract infections (eg. sinusitis).

### STAGE 3

Significant unintentional weight loss.
Diarrhoea for more than a month.
Fever for more than a month.
Oral thrush/candida.
Pulmonary TB in the last year.
Severe pneumonia or other bacterial infections.
Vaginal candida for more than one month, or poor response to therapy.

### STAGE 4

Chronic weight loss plus diarrhoea or fever.
Diagnosed opportunistic infection.
Extra-pulmonary TB.
Kaposi's sarcoma.
HIV dementia.
Diagnosed cancer (eg. Lymphoma).

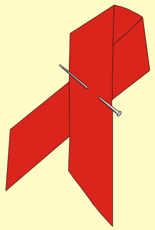

## AUTHORS

Dr R. English
Professor E. Bateman
Dr M. Zwarenstein

UNIVERSITY OF CAPE TOWN
LUNG INSTITUTE

## CONTRIBUTORS

Members of the PALSA project.

MEDICAL RESEARCH
COUNCIL

## ACKNOWLEDGEMENTS

Professor S. Naidoo for the use of the images of oral candida.
Professor C. Prescott for the use of the images of the various ear conditions.

WORLD HEALTH ORGANISATION

*Guideline design* ***Imago -Visual***
